# Maternal Exposure to Arsenic and Its Impact on Maternal and Fetal Health: A Review

**DOI:** 10.7759/cureus.49177

**Published:** 2023-11-21

**Authors:** Nancy Y Ortiz-Garcia, Anayansi Ixchel Cipriano Ramírez, Karen Juarez, Jazmin Brand Galindo, Gabriela Briceño, Ernesto Calderon Martinez

**Affiliations:** 1 General Medicine, Universidad Juárez del Estado de Durango, Durango, MEX; 2 General Practice, Instituto Politécnico Naciónal, Mexico City, MEX; 3 Infectious Disease, Universidad Nacional Autónoma de México (UNAM), Mexico City, MEX; 4 Palliative Care, Instituto Mexicano del Seguro Social, Guadalajara, MEX; 5 Obstetrics and Gynecology, Universidad de Oriente, Barcelona, VEN; 6 Biomedical Informatics, Universidad Nacional Autónoma de México (UNAM), Mexico City, MEX

**Keywords:** atomic absorption spectrometry (ass), arsenite methyltransferase (as3mt), glucocorticoid receptor (gr), interleukins (il-1 and il-8), reactive oxygen species (ros), dimethylarsinic acid (dma), monomethylarsonic acid (mma), glutathione (gsh), s-adenosyl methionine (sam), arsenic (as)

## Abstract

Arsenic exposure is a significant public health issue, with harmful effects caused by its use in commercial products such as car batteries, pesticides, and herbicides. Arsenic has three main compounds: inorganic, organic, and arsine gas. Inorganic arsenic compounds in water are highly toxic. The daily intake of arsenic from food and beverages is between 20 and 300 mcg/day. Arsenic is known for its carcinogenic properties and is classified as a human carcinogen by different institutions. Exposure can lead to oxidative stress, DNA damage, and epigenetic deregulation, which can cause endocrine disorders, altered signal transduction pathways, and cell proliferation. In addition, arsenic can easily cross the placenta, making it a critical concern for maternal and fetal health. Exposure can lead to complications such as gestational diabetes, anemia, low birth weight, miscarriage, and congenital anomalies. Female babies are particularly vulnerable to the negative impact of arsenic exposure, with a higher risk of low weight for gestational age and congenital cardiac anomalies. Therefore, it is crucial to monitor and regulate the levels of arsenic in drinking water and food sources to prevent these adverse health outcomes. Further research is necessary to fully understand the impact of arsenic exposure on human health, especially during pregnancy and infancy, by implementing preventative measures and monitoring the levels of arsenic in the environment.

## Introduction and background

Arsenic (As) exposure is a transcendent and barely known issue that causes many harmful health effects [1,2]. Arsenic and its chemical compounds have been produced for commercial purposes for centuries, being used worldwide. It is used in car batteries, pesticides, wood preservatives, glass, pharmaceutical, electronic, ammunition, and dye factories. From a biological and toxicological perspective, there are three main groups of arsenic compounds: inorganic, organic, and arsine gas [1-4]. Inorganic arsenic is found in the environment combined with other elements such as oxygen, chlorine, and sulfur. Organic arsenic is found combined with carbon and hydrogen [5].

Contamination through food depends on the levels of arsenic in water and soil to which these foods are exposed [6]. Inorganic arsenic compounds in water are highly toxic, while organic compounds, such as those in seafood, fruits, and vegetables, are less harmful to health (Table 1) [7-9]. Thus, the primary arsenic exposure is ingesting contaminated food or water. Nevertheless, it can be linked to polluted air, soil, and occupational exposure, the last one mostly for inhalation of arsenic-containing particles [9,10]. The daily intake of total arsenic from food and beverages in the global population is generally between 20 and 300 mcg/day [7].

**Table 1:** Sources and routes of exposure to arsenic Data from references [11,12]

Main sources	Sources	Route of exposure
Dietary Sources	Seafood (algae, detritivorous fishes, marine fish, and invertebrates), groundwater, fruits, and grains.	Ingestion
Earth Crust	Soils, rocks, volcanism, natural fires, hydrothermal mineral deposits, geothermal systems and marine spray, metal mining.	Ingestion/Inhalation
Industrial Sources	Wood preservatives, burning fossil fuels, glass, enamels, paints, fabrics, leather manufacturing, medical waste.	Ingestion/Inhalation
Agricultural sources	Pesticides, herbicides, insecticides, feed additive (Poultry/chicken).	Inhalation/Ingestion
Air	Pesticides, herbicides.	Inhalation

The first chemical whose carcinogenic characteristics were recognized was As. In 1947, Neubauer concluded that the inhalation of arsenic caused lung cancer in the studied subjects [13,14]. Since then, arsenic has been classified as a human carcinogen by the Department of Health and Human Services and the International Agency for Research on Cancer. In certain countries, groundwater contains arsenic naturally. Due to this, arsenic is an established carcinogen and the most crucial drinking water contaminant [10,15].

In 2001, the World Health Organization (WHO) adopted a new limit on overall total arsenic levels in drinking water, 10mcg/l; previously, the permissible limit was 50 mcg/l. Although the suggestion to further lower the limit to 2 mcg/l was made, the proposal was rejected due to financial implications [14]. In Mexico, the maximum amount of arsenic authorized in drinking water is 50 mcg/l, with a progressive reduction to 25 mcg/l in 2005 [16]. Even with the adjustment, the limit is higher than the WHO limit recommended. As a result, drinking water with inorganic arsenic contamination is a global public health issue, this does not mean that this is the daily tolerable drinking water limit, it is a permissible lever in water overall [10,17,18]. There are several consequences according to different arsenic concentration exposures (Table 2). The short- and long-term effects of arsenic on adults have been studied in detail [4,19-33]. However, little interest has been placed on the consequences of arsenic exposure during pregnancy, childhood, and adolescence [9,34,35]. Moreover, arsenic has a spectrum of non-carcinogenic chronic health effects, including adverse reproductive outcomes such as abortion, pregnancy losses, low birth weight, and congenital malformation [36]. All of these are water arsenic level related. More information and understanding of its effects on the mother-child pair are essential. This review paper aims to describe arsenic’s effect on the mother-child pair.

**Table 2:** Consequences and disorders linked to different arsenic concentration exposure levels Data from references [37-45]

Disorder/Consequence	Arsenic Concentration Exposure
Miscarriage/Stillbirth	>100 mcg/L
Gestational Diabetes	>50 mcg/L
Anemia during Pregnancy	>50 mcg/L
Low Birth Weight	>50 mcg/L
Increased Systolic Blood Pressure in Pregnancy	20-50 mcg/L
Congenital Heart Anomalies in Female Newborns	>10 mcg/L
Passive Muscle Tone in Newborns	0.73 mcg/L
Behavioral Ability in Newborns	0.73 mcg/L
Infant Mortality	>555 mcg/L creatinine

## Review

Damage mechanism

**Figure 1:**
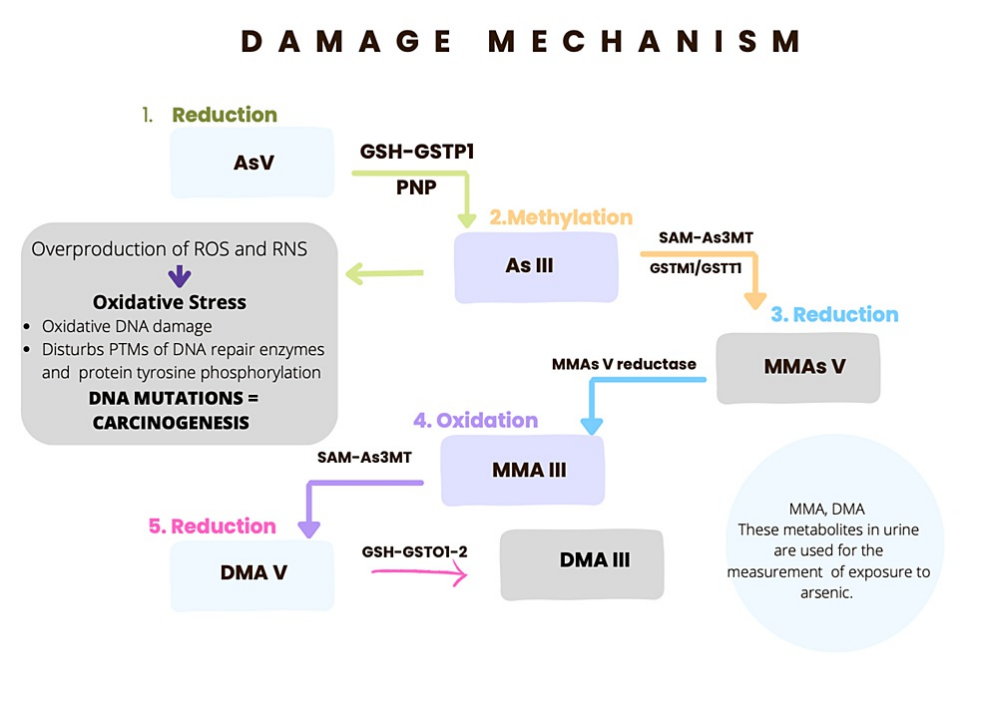
Damage mechanism and methylation of arsenic 1. The gastrointestinal tract absorbs the As V. Here, it passes to glutathione (GSH)-mediated two-electron reduction and converts to As III. 2. As III goes through oxidative methylation by arsenite methyltransferase (As3MT), it becomes pentavalent organic arsenic species (Monomethylarsonic acid V, MMAs V) in the liver. As III produces ROS, these alter the DNA structure by inserting or deleting multiple mutations in tumor suppressor genes, which can lead to cancer formation if it’s not repaired. 3. The MMA V can be methylated and generate trivalent arsenic compounds, such as MMA III and dimethylarsinic acid (DMA) III. RNS: Reactive nitrogen species; PTMs: post-translational modifications; PNP: purine nucleoside phosphorylase; GSTO: glutathione S-transferase omega 1; GSTT1: glutathione S-transferase theta 1; GSTM1: glutathione S-transferase Mu 1; SAM: S-adenosyl methionine [20,21]. Image credits: Anayansi Ixchel Cipriano Ramírez.

The mechanism depends on the site, tissue, dose, metabolism, chemical form, route, and duration of exposure [21,46]. There is evidence suggesting that inorganic arsenic is extensively methylated. Arsenic methylation occurs in an oxidative methylation reaction in which trivalent forms of arsenic are sequentially methylated to form mono-, di-, and trimethylated products using S-adenosyl methionine (SAM) as the methyl donor and glutathione (GSH) as an essential co-factor [47,48]. Metabolites are primarily excreted in the urine. Dimethylarsinic acid (DMA) and monomethylarsonic acid (MMA) are metabolites that were recently identified in urine and environmental materials. DMA accounts for 60-80% of the total metabolites, while MMA accounts for the remaining 10-20%. This discovery suggests that methylation of inorganic arsenic is a widespread phenomenon, thus allowing both metabolites to be used as arsenic exposure indicators [49,50]. The DMA V was reported as a teratogen, a nephrotoxin, and a complete carcinogen in mammals (Figure 1) [20,21].

**Figure 2:**
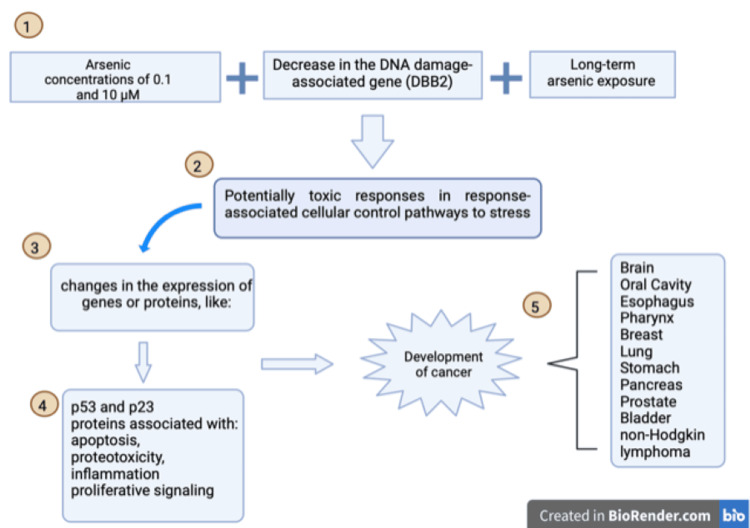
Arsenic damage pathway 1. The duration and exposure are the main issues of arsenic damage in DNA. 2. This is conducted to lower ineffective DNA repair mechanisms and increase DNA injury. 3,4. The main arsenic cell damage pathway is with p53, which dysregulation leads to apoptosis, proteotoxicity, inflammation, and proliferative signaling.  5. These pathways' disruption in the cell cycle causes cell cycle progression without an important mutation checkpoint, leading to numerous cancers. Image Credits: Jazmin Brand Galindo (Created with BioRender.com) [55]

The damage mechanism includes events that affect multiple cellular processes, generating oxidative stress and producing reactive oxygen species (ROS) [20,51]. Arsenic has been found to cause cell arrest at the G1 or G2-M phase. However, the exact chemical mechanism is unknown and has been linked to the main signaling system, the p53 pathway [20]. In addition, oxidative DNA damage, inhibiting DNA damage repair mechanisms and chromosomal and genomic instability, alters epigenetic regulation and generates deregulation of cell proliferation (Figure 2). All these damage mechanisms are closely related to the development of endocrine disorders and alteration signal transduction pathways [49,52,53]. All this acquires relevance in terms of health, especially maternal and fetal, due to the ease of arsenic crossing the placenta, making the pregnancy critical [54-57].

Clinical approach

Signs and symptoms of arsenic toxicity depend on the type of arsenic, exposure route, and whether acute, subacute, or chronic (Table 3) [7,10,32].

**Table 3:** Signs and symptoms of acute, subacute, or chronic arsenic poisoning Data from references [27,28]

Classification	Time	Symptoms
Acute arsenic poisoning	After 15 hours and survived 48 hours	Vomiting, abdominal pain, diarrhea followed by numbness in the extremities, muscle cramps, and in extreme cases, death
Subacute arsenic poisoning	After 5–8 days of continuous arsenic ingestion	Gastrointestinal symptoms, diarrhea, headache, vertigo, weakness, and facial edema
Chronic arsenic poisoning	Long-term exposure	Cardiovascular disease, peripheral vascular disease, lung disease, diabetes mellitus 2, anemia, hepatotoxicity, nephrotoxicity, immunotoxicity, neurotoxicity, and developmental defects. Skin, lung, liver, bladder, kidney, breast, stomach, pancreas, brain, prostate, oral cavity, pharynx, esophagus, and colorectal cancers, and non-Hodgkin lymphoma.

Symptoms of acute arsenic poisoning include vomiting, abdominal pain, diarrhea followed by numbness in the extremities, muscle cramps, and in extreme cases, death [28].

Subacute arsenic poisoning, in which an extremely high dose of arsenic was ingested in the short term, initially presented with gastrointestinal symptoms after 5-8 days of continuous arsenic ingestion [10,22,27,28,58]. Though diarrhea was predominant, headache, vertigo, weakness, and facial edema were the main complaints besides the gastrointestinal symptoms. Only 24% of patients with gastrointestinal symptoms exhibited facial edema, whereas 43% of patients without gastrointestinal symptoms had facial edema [27]. 

Chronic arsenic poisoning is due to environmental or occupational exposure and has a more insidious onset. Long-term exposure to arsenic is associated with cardiovascular disease, peripheral vascular disease, lung disease, diabetes mellitus 2, anemia, hepatotoxicity, nephrotoxicity, immunotoxicity, neurotoxicity, and developmental defects [18,59].

Several mechanisms have been described over the years to explain cancer development, as described above (Figures 1, 2) [55]. The response to an arsenic concentration of <0.1 μM indicated adaptive responses but also some significant changes, such as a decrease in the DNA damage-associated gene (DBB2) associated with the maintenance of DNA integrity. Exposures to concentrations of 0.1 and 10 μM resulted in potentially toxic responses in response-associated cellular control pathways to stress, changes in the expression of genes or proteins p21 and p53, as well as changes in the face of genes or proteins associated with apoptosis, proteotoxicity, inflammation, and proliferative signaling [7,20,33,60]. Long-term arsenic exposure is related to cancer development, as mentioned above (Table 3) [19,61,62].

Arsenic-contaminated water is associated with an increased risk of bladder and kidney cancer. Research has suggested that exposure to at least 10 mcg/L of arsenic in drinking water doubles the risk of bladder cancer or increases the risk by forty percent [63]. It has been shown that there is a clear dose-response relationship between arsenic water level and bladder, kidney, and lung cancers [64]. Inhaled inorganic arsenic increases the risk of lung cancer, affecting mostly workers in mines and industrial and chemical factories [23]. 

Recent evidence suggests an association between populations exposed to high levels of inorganic arsenic in drinking water and the development of bladder cancer. Therefore, the WHO recommended a limit for the concentration of arsenic in drinking water from 25 mcg/L to 10 mcg/L. However, populations exposed to inorganic arsenic concentrations as low as 10 mcg/L in drinking water have been shown to have a 40% increased risk of bladder cancer compared to the unexposed populations. This makes it a substantial public health problem, and the permissible limit for arsenic concentration in drinking water needs to be addressed appropriately [23].

Dermatological manifestations 

Skin lesions are the most common clinical manifestations of chronic arsenic exposure and positively correlate with arsenic levels in water. They occur after at least five years and can lead to skin cancer [7,65]. This explains why skin lesion rates were more significant among elderly people than younger people because the elderly have fewer efficient methods to metabolize As [65]. Also, these lesions are more frequent in males than females. Pigmentary changes are the earliest cutaneous changes of chronic arsenic exposure, including hyperpigmentation lesions of the chest with raindrop hypopigmentation, palmar, and plantar hyperkeratosis, spotted melanosis, leukomelanosis, dyschromia, and mucosal pigmentation [66]. The most common sites are the nipples, axilla, palms, and pressure points [24].

Recent data of long-term, low-level exposure to inorganic arsenic in humans have reported the presence of these lesions, which began to manifest at chronic exposure levels >0.02 mg As/kg/day [5]. 

Ingestion of inorganic arsenic increases the risk of developing Bowen’s disease and squamous and basal cell carcinoma [67,68]. A peculiarity of skin cancer due to arsenic is the localization on sun-protected skin [69].

The mechanism of the skin effect is the lower arsenic methylation capacity and reduced arsenic metabolism [65]. Arsenic binds to keratin and accumulates in skin, hair, nails, and mucosae. It was found that the keratin types Krt1 and Krt10 are both markers of hyperkeratosis and are found in keratinizing squamous cell carcinoma caused by arsenic [25]. Also, As alters the levels of interleukins (IL-1 and IL-8), granulocyte-macrophage colony-stimulating factor, and transforming growth factor- β in conjunction with the damage mechanisms mentioned in previous sections [24].

Arsenic and placenta 

The placenta maintains pregnancy and controls the transfer of gases and nutrients between mother and fetus, which is essential for fetal growth and development [70,71]. Exposure to harmful or toxic elements in preconception during the first trimester can lead to structural alterations when compromised organogenesis [71,72]. Arsenic accumulates up to three times the level in maternal blood, and its level in fetal cord blood is similar, 9.2 mcg/liter, almost as high as in maternal blood just before delivery. This demonstrates that arsenic is easily transferred across the placenta to the fetus [73]. There is evidence that for every microgram per liter of arsenic present in household water, there is a 2.1% increase in placental arsenic deposits [54]. 

Inorganic arsenic and methylated metabolites can cross the human placenta [53], increasing the risk of miscarriage, stillbirth, poor fetal growth, and increased infant death rate [35]. Evidence suggests an apparent increase in fetal loss and premature delivery in the women with the highest concentrations of arsenic in their drinking water due to abnormal placental vasculogenesis and placental insufficiency due to toxic effects mediated via oxidative stress, ineffective transport of nutrients to the fetus may lead to placental pathology and preeclampsia [54,74,75]. Maternal exposure to arsenic increases oxidative stress and inflammation in the placenta; in early pregnancy, the arsenic reduces T-cell numbers in the placenta, causing a disruption of immune balance and increasing the risk factors for infectious diseases [76]. Inorganic arsenic has an endocrine disruptor in the placenta because the MMA and DMA induced expression of eight glucocorticoid receptor (GR)-associated genes, decreasing DNA binding of this GR [77]. This receptor is essential for proper placentation and fetal development [77].

Effects on maternal health

Pregnancy entails physical, anatomical, and physiological changes. These changes include the progressive increase of plasma volume, increased production of thyroxine-binding globulin, aldosterone, prolactin, and oxytocin, and, most importantly, an increase in progesterone to maintain a viable pregnancy [78-81]. The adaptive changes in pregnancy affect all the body's systems and include a progressive increase in plasma volume as the pregnancy progresses. Many of these physiological adaptations are due to increased estrogen, progesterone, prolactin, relaxin, human placental lactogen, oxytocin, and human chorionic gonadotropin, contributing to changes required for the growing fetus [82]. Arsenic interacts with steroid hormones and estrogen, causing adverse effects on maternal health, child development, and women's health in general [53]. More than 50 mcg/l of arsenic in groundwater had been associated [53].

Pregnant women exposed to >100 mcg/L arsenic are more likely to experience miscarriage and stillbirth [9]. An association has been found between high concentrations of arsenic (more than 50 mcg/L) with complications during pregnancy, such as gestational diabetes, anemia, and low birth weight [10,38]. Exposure to low concentrations of arsenic in drinking water (20-50 mcg/L) is a risk factor for increased systolic blood pressure in women during and after pregnancy [39,40]. The relationship between arsenic and pre-eclampsia can only be explained as related. Cross-sectional studies have reached conflicting conclusions and require further investigation [37,38]. The burden of cardiovascular disease attributable to arsenic in drinking water significantly impacts young and healthy women [38,39,83]. In a Bangladesh cohort, there was no clear evidence for an effect of arsenic exposure during pregnancy, on postpartum insulin resistance or beta cell function, in contrast to what has been described in the literature [84]. 

Effects on fetal health 

The fetus is more exposed to inorganic arsenic and MMA in early gestation before maternal metabolism. Methylation is inhibited at high levels of arsenic exposure, and the fetus will be exposed to more inorganic arsenic and MMA. Fetus exposed to arsenic in-utero presented congenital anomalies, concluding a positive association between the risk of suffering congenital anomalies, specifically congenital heart anomalies, in females with more than 10 mcg/l of arsenic exposure in drinking water [9,42,43,56,85,86]. This association was not found in the male sex. Therefore, more studies are needed to find an explanation related to the female gender [87].

Arsenic exposure has been implicated in hypoxia and the generation of oxidative stress, interrupting the typical plantation, adversely affecting fetal growth, causing low birth weight, and increasing the risk of preterm birth and low weight for gestational age [38,88-90]. Increased exposure to arsenic may impact biological functions in the fetal placenta, a sex-dependent subset. Female newborns are more vulnerable to arsenic-induced birth-weight reduction that may involve the activation of stress response pathways in the placenta [60,72].

Prenatal arsenic exposure was inversely related to neurobehavioral development in neonates, particularly for behavioral ability and passive muscle tone. These findings were more noticeable in older mothers [44]. There is evidence that the effects reported were observed at relatively low concentrations of arsenic, 0.73 mcg/l of umbilical cord serum [44]. Moreover, the potential connection between prenatal exposure to arsenic and domain-specific development in six-month-old infants is investigated. A recent study found that moderate and high levels of arsenic were significantly linked to a greater risk of presumed developmental delay compared to low levels. Additionally, exposure to low levels of arsenic in the womb may adversely affect the domain-specific development of female infants [87].

Predictors

As already mentioned, MMA and DMA are the primary metabolites in urine, which are used as indicators of exposure to inorganic arsenic and as a measure of the efficiency of its metabolism. Atomic absorption spectrometry is used to analyze urine samples and determine the presence of MMA and DMA. Although the cost per test is relatively low, acquiring the equipment can be quite elevated, making it inaccessible to the countries with higher arsenic concentrations in water since these countries tend to be among the poorest. Another limitation is that each element requires a specific atomization temperature, making the process less efficient and costly if multiple elements are tested simultaneously [91,92]. Relationships between arsenic levels in urine samples, fetal development, and neonatal birth outcomes have been studied and verified. Arsenic metabolites are excellent predictors of fetal growth and neonatal delivery [9,35,36,42,43,85,93]. Arsenic metabolite levels in urine were negatively correlated with fetal thoracic circumference during the first trimester, fetal head diameters in all the trimesters, and low weight at birth [85]. Other studies have confirmed that arsenic concentrations in maternal urine samples were associated with low birth weight, lower mean gestational age, and shorter newborn length measurements [42,43,94]. These characteristics are only shown in female newborns and are most noticeable in the third trimester [9,42,43,85,90,93,95]. It has been established that there is a negative correlation between the level of arsenic in maternal urine during pregnancy, fetal development, and pregnancy outcomes [85].

Arsenic and new-born mortality

Strong evidence exists linking prenatal exposure to arsenic with adverse pregnancy outcomes and increased neonatal mortality rates. Specifically, research has found a significant correlation between moderate to high levels of exposure to arsenic during pregnancy (exceeding 555 mcg/l creatinine) and a heightened risk of stillbirth and infant mortality [34]. Additionally, there is an increased risk of infant mortality when arsenic exposure is greater than one mcg/l in this population [96].

There is strong evidence that neonatal mortality increased due to arsenic exposure in both the mother and fetus [9,34,86]. Two investigations found a negative relationship between arsenic exposure and neurobehavioral development, behavioral delay, behavioral ability, low muscle tone, and domain-specific development in girls at six months of age that can lead to increased mortality and reduced life years [87]. More evidence concluded that maternal and in-utero exposure to arsenic increases the risk of infections during the first year of life, particularly diarrhea and respiratory symptoms [40]. Additionally, male infants were observed to have increased length and decreased head and chest circumference, later confirmed by another study, and found to persist for the first year of life [42,43,85,97].

## Conclusions

Arsenic exposure's impact on human health is complex and far-reaching. The damage mechanism is influenced by several variables, such as location, tissue type, dosage, metabolism, chemical form, exposure route, and duration. Inorganic arsenic often undergoes methylation, forming metabolites. These metabolites disrupt cellular processes, leading to oxidative stress, DNA damage, and disturbances in cell proliferation and signaling pathways. These changes contribute to the development of endocrine disorders and carcinogenesis. Clinical manifestations of arsenic toxicity vary with exposure type and duration. Acute arsenic poisoning can result in symptoms like vomiting, abdominal pain, and, in severe cases, death. Chronic exposure is associated with a range of health issues, including cardiovascular and lung diseases, as well as various cancers. Chronic arsenic exposure often manifests as skin lesions and skin cancers, especially in regions with contaminated water sources.

Arsenic exposure during pregnancy poses significant risks to both mothers and fetuses. Pregnant women exposed to arsenic may experience complications like miscarriage, stillbirth, gestational diabetes, anemia, low birth weight, and pre-eclampsia. Fetal exposure to arsenic can lead to congenital anomalies, low birth weight, preterm birth, and developmental delays. The placenta facilitates the transfer of arsenic to the fetus, particularly impacting female infants and older mothers. Arsenic metabolite levels in maternal urine can predict adverse pregnancy outcomes and neonatal health. High arsenic exposure during pregnancy is associated with increased neonatal mortality, especially at elevated exposure levels. In summary, arsenic exposure profoundly affects maternal and fetal health, resulting in developmental delays, congenital anomalies, and higher mortality rates. This underscores the need for rigorous monitoring and regulation of arsenic levels in drinking water and other sources to safeguard public health.
